# An energy harvesting shock absorber for powering on-board electrical equipment in freight trains

**DOI:** 10.1016/j.isci.2023.107547

**Published:** 2023-08-05

**Authors:** Shengxin Wang, Wumao Peng, Weihua Kong, Dabing Luo, Zutao Zhang, Longfei Li

**Affiliations:** 1 School of Mechanical Engineering, Southwest Jiaotong University, Chengdu 610031, P.R. China; 2 Yibin Research Institute, Southwest Jiaotong University, Yibin 64000, P.R. China; 3 Tribology Research Institute, State Key Laboratory of Traction Power, Southwest Jiaotong University, Chengdu 610031, P.R. China

**Keywords:** Applied sciences, Electrical system, Energy storage

## Abstract

To realize smart detection and safe operation of freight trains, a continuous and stable energy source is required for electrical equipment on the train. It is a feasible scheme to harvest the vibration energy of train suspension to supply power for on-board electrical equipment. This paper presents an energy-harvesting shock absorber (EHSA) based on the slider-crank mechanism and ratchet-pawl mechanism, which provide a vibration reduction effect and renewable electricity. To determine the damping performance and the power generation performance of EHSA, a dynamic model is established based on MATLAB. According to the tests on the mechanical testing and sensing (MTS) bench, the maximum power generation mechanical efficiency of the EHSA is 67.75%, and the maximum output power of the EHSA is 1.65W. In addition, the charging tests on the MTS bench show that the proposed device is applicable to power on-board electrical equipment on freight trains.

## Highlights

• A vibration energy-harvesting shock absorber is proposed for freight trains
• A reliable and efficient mechanical motion rectifier is proposed
• The maximum mechanical efficiency of power generation was 67.75%
• Experiments with mechanical testing and sensing and charging were conducted

## Graphical abstract

**Figure:**
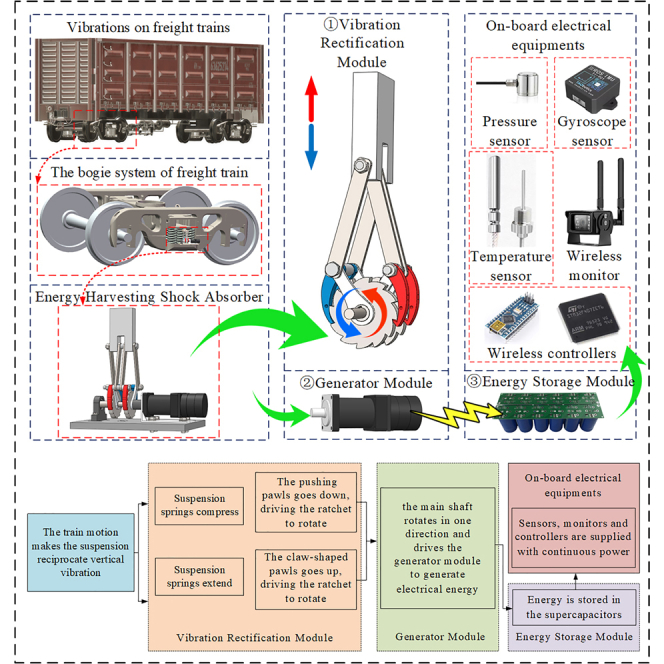

## Introduction

Freight trains are one of the most important modes of modern logistics.^1^ With the widespread applications of Internet of Things (IoT) technology in trains, including train management, operation, maintenance, video monitoring systems and train control systems,^2^ freight trains’ efficiency, economy, and safety are further developed.^3^ Based on the IoT technology, in the daily operation of modern freight trains, a large number of electrical equipment, such as various sensors,^4^ monitors,^5^ and controllers,^6^ are required to monitor and control freight trains to ensure their safe and efficient operation. At present, standard freight trains are only equipped with brake pipes, not electrical pipes,^7^ so the power supply of these on-board electrical equipment mainly comes from chemical batteries. However, due to their limited working life, chemical batteries have become increasingly challenging to meet the energy needs of modern freight trains. Frequent battery replacement will not only waste human and financial resources, but also pollute the environment.^8^ Therefore, the development of an efficient, stable, and sustainable environmental energy harvesting system is a promising solution for the power supply of onboard electrical equipment in freight trains.

There are various forms of energy in the environment around the trains,^9^ including solar,^10^ wind,^11^ and vibration energy.^12^ Various researchers have proposed and designed railway energy harvesters based on various energy conversion mechanisms. Hao et al. designed a miniature solar collector with foldable wings to power low-power equipment on the trackside, which integrates optical and mechanical sensors to control the switch of the wings depending on weather conditions to reduce dust buildup.^13^ Pan et al. proposed a novel structural energy harvesting device, generating electricity with the wind in the tunnel by two specially designed rotors to supply power for low-power sensors inside rail tunnels.^14^ Wang et al. designed a solar-wind hybrid energy harvesting device for the Qinghai-Tibet Plateau. According to the experimental results, when the wind speed is set at 13 m/s, and the load resistance is set at 8Ω, its output power reaches 1.08W.^15^ However, whether solar energy, wind energy, or a combination of the two, their energy harvesting effect depends on the weather and is unstable. This is contrary to the energy requirements in the modern railway system and may affect the safety of trains in extreme cases. Therefore, the vibration energy around the railway has attracted the attention of researchers due to its universality.

The vibrations in the railway system do not depend on the environment and weather and are widespread and stable energy sources. Usually, this vibration energy is eventually transformed into heat energy and dissipated.^16^ In the existing research, environmental vibration energy harvesters can be divided mainly into piezoelectric and electromagnetic systems.^17^

Based on the piezoelectric mechanism, piezoelectric energy harvesters generate electricity by absorbing and converting mechanical movements,^18^ with high sensitivity, service life, and energy density.^19^ Gao et al. developed a piezoelectric cantilever beam to increase the strain in rail deflection and improve the power generation performance. Tests showed that its peak power was 4.9 mW, enough to power low-power appliances.^20^ In order to solve the problem of insufficient power of piezoelectric transducers, Yuan et al. used an array of drum-type piezoelectric transducers placed under sleepers, whose power generation capacity under the real rail can reach 100 mW.^21^ Wang et al. proposed using piezoelectric transducers in a stacked and patched arrangement to harvest vibrations from the rail and generate electricity for sensors to monitor the train’s real-time operation status.^22^

On the other hand, based on the electromagnetic conversion mechanism,^23^ vibration energy generators in this field consist of linear and rotary types. Gao et al. first developed a rail energy harvester based on the linear electromagnetic generation mechanism with a peak output voltage of 2V.^24^ Later, Gao et al. designed a prototype of an energy harvester based on the rotary electromagnetic power generation mechanism, which is used to harvest the vibration energy of wheel and rail and integrates the charging management system and wireless sensor nodes (WSN).^25^ Compared with the linear electromagnetic generator, the rotating electromagnetic generator has a more compact structure and is more suitable for layout in the railway system. Meanwhile, their higher energy density can support larger-scale WSNs, monitors, and controllers in the future.^26^ However, most of the energy harvesters in the existing research are designed to supply power to the equipment along the track, ignoring the electricity demand of the on-board electrical equipment.

Currently, the vibration energy harvesting (VEH) technology in railway systems can still be improved from these three aspects: (1) optimizing the structure, size, and layout of the vibration energy harvester to improve its stability, durability, and economy, (2) making the energy-harvesting shock absorber (EHSA) universal in the field of a freight train, easy to assemble and disassemble, and not changing the structure of the original train, and (3) improving the mechanical efficiency and generating power of vibration energy generator to supply power for a wider variety of on-board self-powered equipment.

In this study, an EHSA based on the slider-crank mechanism and ratchet-pawl mechanism is designed, and its modeling, simulation, in-lab tests, and field installations are carried out. The kinematics model of vibration rectification mechanisms and the dynamics model of the system is developed to predict generator speed and system damping under different excitation, and simulation experiments are carried out as preliminary verification. In the MTS bench test, the sinusoidal displacements with the amplitude of 7mm and frequencies of 1–3.5mm are used as input, and the highest power output of 1.65W can be obtained through EHSA, and the maximum mechanical efficiency of 67.75% is achieved. Compared with the existing on-board electromagnetic train energy harvesters, the proposed EHSA has the advantages of small size, simple manufacture, reliable transmission, excellent power generation performance and easy installation on different types of freight train suspensions. In practical application, the EHSA is installed on the train suspension via the clamps, used to harvest the vibration energy of the train suspension to supply power for the on-board equipment on the freight train. When freight trains are traveling at a certain speed, the suspensions of the trains will continuously vibrate vertically. The EHSA converts vibration energy into electrical energy. After rectification, voltage regulation, and storage, it is used to supply power for on-board electrical equipment such as sensors, monitors, and controllers on freight trains. With the help of the IoT, these devices can feed real-time information about trains to a remote monitoring system and, if necessary, carry out corresponding instructions to avoid severe accidents. As the scale of on-board electrical equipment continues to expand, the use of battery power will lead to maintenance difficulties, waste of human and material resources, and environmental pollution problems. On the contrary, the vibration energy of the train suspension always exists with the train operation. Through the presented EHSA in this paper, a steady stream of electricity will be provided to the equipment on the train. Therefore, the research of the EHSA for freight trains in this paper is highly beneficial to the environment and energy.

The rest of the paper is arranged below. The section “system design” introduces EHSA, including vibration rectification, generator, and energy storage modules. The section “modeling and analysis” elaborates on the modeling and theoretical analysis of the presented EHSA, mainly including kinematic analysis of the vibration rectification mechanism, dynamic analysis, and simulation testing of the system. The section “bench test details” describes the details of the experiments of the generation performance and efficiency of EHSA. The section “experimental results and analysis” discusses the results of the experiments. The section “conclusions” illustrates the conclusion of the paper. Finally, the section “limitations of the study” points out the existing problems of the study and the directions of further research.

### System design

**Figure 1:**
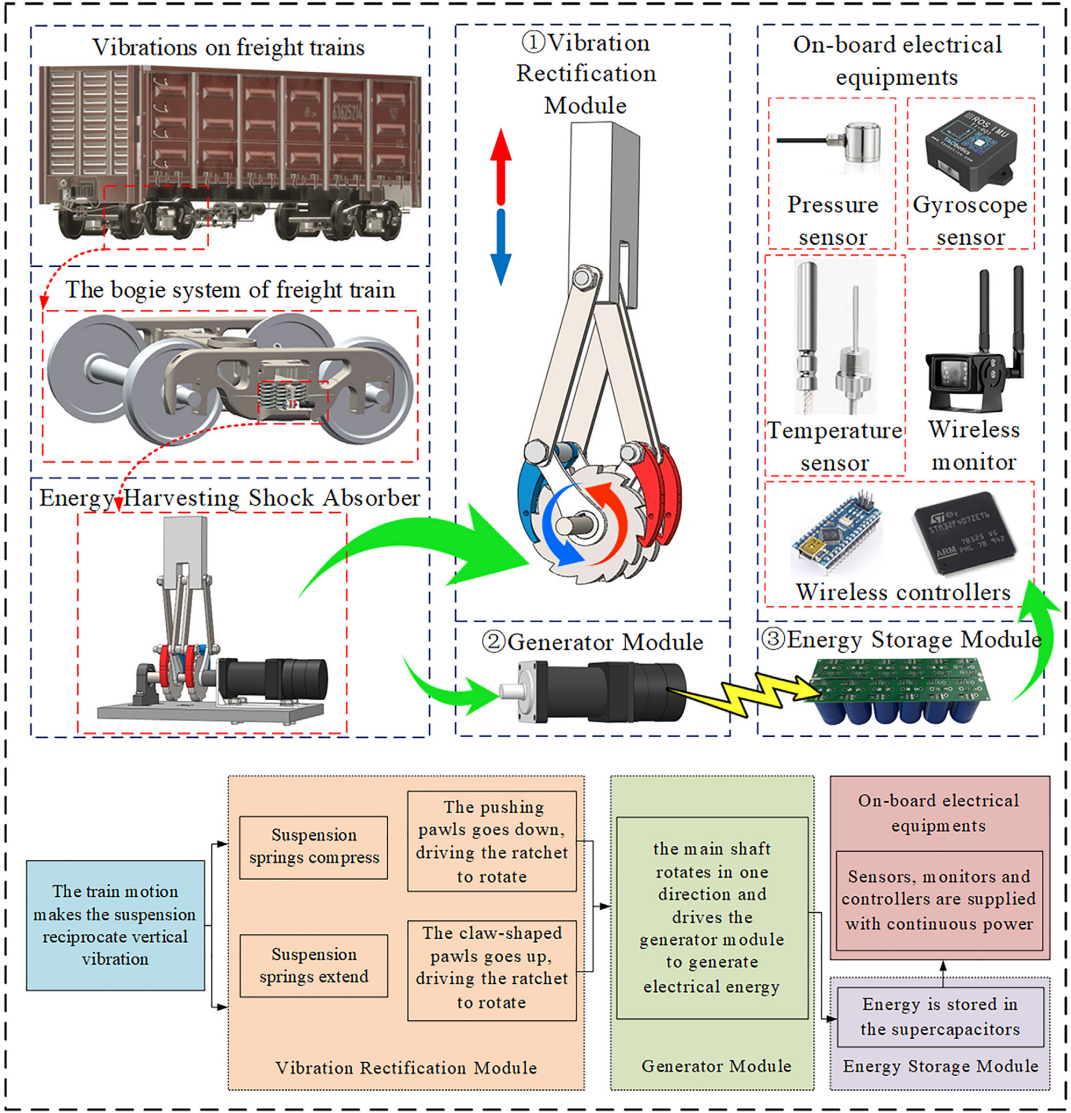
System design of the EHSA

The EHSA presented in this paper is mainly composed of three modules —vibration rectification, generator, and energy storage module— and its complete architecture is shown in Figure 1. The primary function of the EHSA is to harvest the vibrations generated by freight train suspensions and provide damping force to reduce the vibration amplitude of the suspensions. During the movement of the freight train, the reciprocating vertical vibration generated by its suspension is transmitted to the EHSA. These vibrations have a strong, rapidly changing impact force and cannot be directly input into the generator module. The mechanical motion rectifier (MMR) can reduce the impact force in train vibration and improve the reliability and durability of the EHSA. In addition, the bidirectional vibration of the train suspension is converted into the unidirectional rotation of the generator by the MMR, which also eliminates the inertia loss when the rotating bidirectional switching and further improves the power generation efficiency. Therefore, the MMR-based vibration rectification module is essential in EHSA systems. The vibration rectification module rectifies the bidirectional vibrations into one-way rotary motion through the slider-crank mechanism and the ratchet-pawl mechanism, and the rectified motion will serve as the power source for the generator module. Then, with the one-way rotary motion, electricity is generated from the generator module. The output energy is stored in supercapacitors of the energy storage module, which supplies power for various electrical equipment on the freight train, such as on-board sensors, monitors and controllers.

#### Vibration rectification module

**Figure 2:**
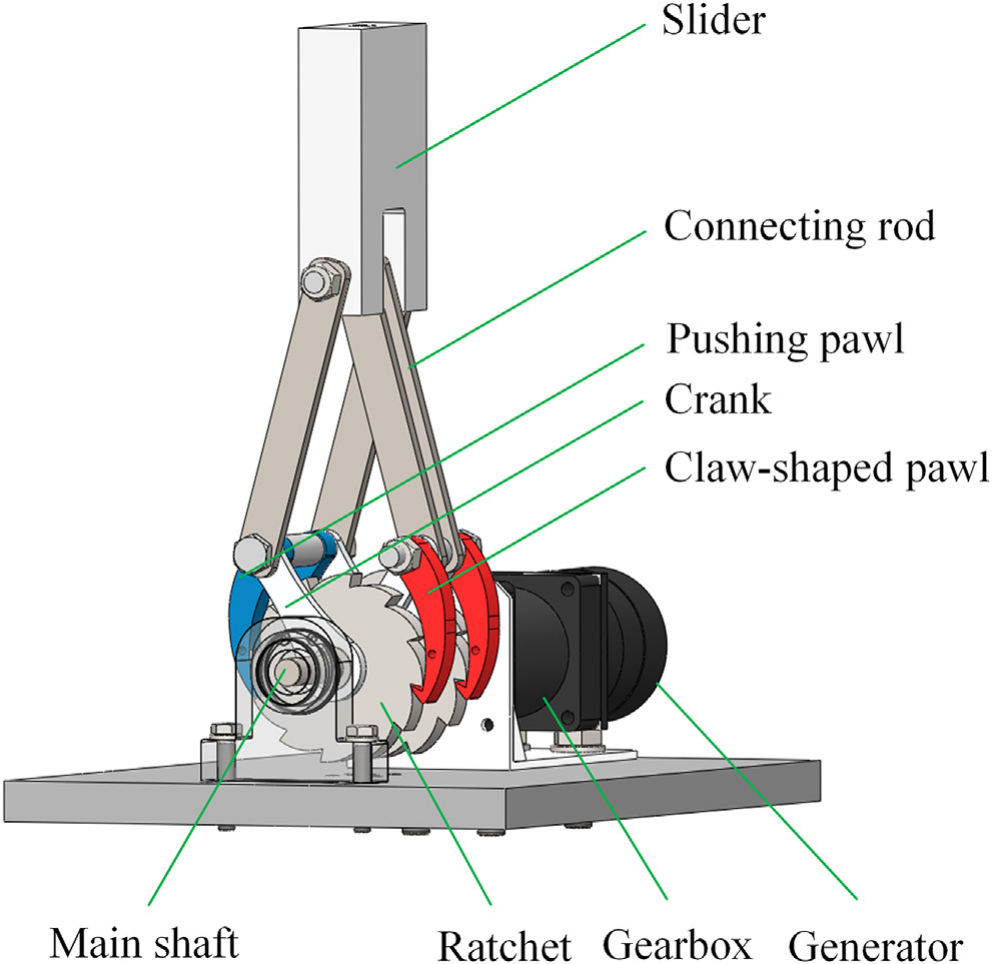
Main structure of the EHSA

**Figure 3:**
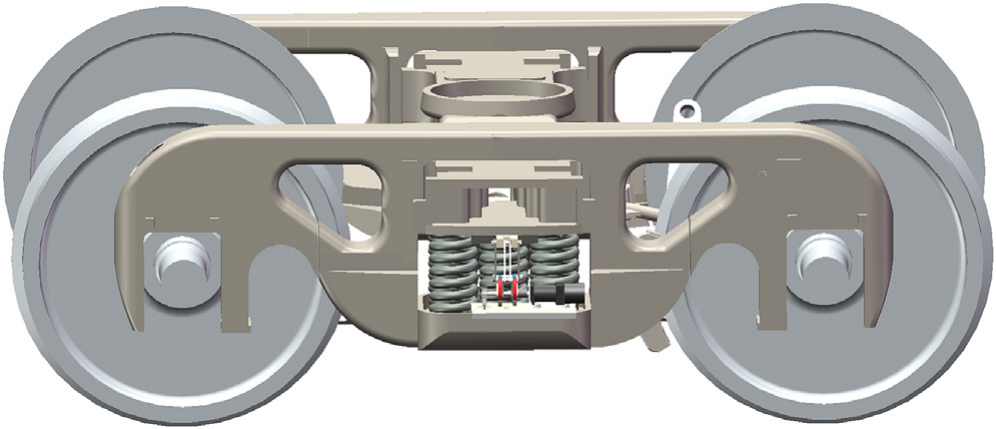
The layout of the EHSA on the bogie of freight trains

Figure 2 shows the structural design of the EHSA, and the vibration rectification module is its core component, composed of the slider-crank mechanism and ratchet-pawl mechanism. The specially designed double-cranks structure can absorb bidirectional vibrations. Moreover, the specially designed double-pawls structure can rectify bidirectional vibrations into one-way rotations. To capture the suspension vibrations, the EHSA was mounted on freight train suspensions, parallel to the coil spring, as shown in Figure 3. The slider is fixed on the top of the spring, the connecting rod and the crank are connected by bolts, and the transmission between the ratchet and the main shaft is accomplished through a flat key connection. One end of the main shaft is mounted on the base plate by the bearing and bearing seat, and the other is connected to the generator module, which is installed on the base plate through a special bracket.

**Figure 4:**
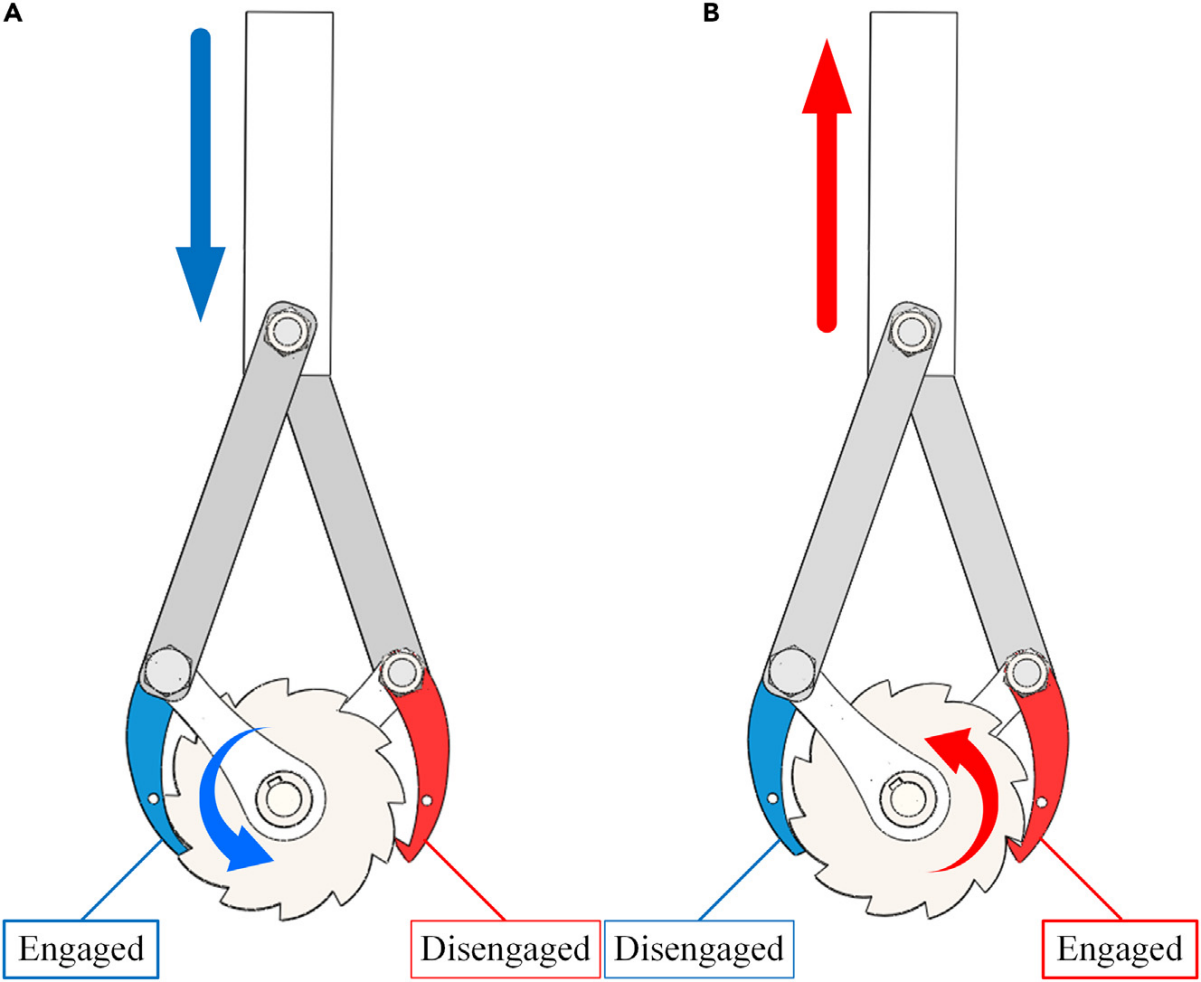
Motion process of the EHSA (A) Compression stroke. (B) Extension stroke.

When the train is in operation, the telescopic motion of the suspension is absorbed by the vibration rectification module by the fixed devices of the slider and the base plate. The vibration rectification module rectifies the bidirectional linear motion into one-way rotary motion, and the resulting one-way rotary motion is then transferred to the generator module. The detailed motion rectification demonstration of the vibration rectification module of the EHSA is shown in Figure 4.

Figure 4A demonstrates the compression stroke of the EHSA. When the suspension compresses, the slider at the top moves vertically downward and drives the connecting rods and cranks to rotate. At the same time, the pawls attached to the crank and arranged on either side of the ratchet move down. The claw-shaped pawls slide down the edge of the ratchet without transmitting any motion to the ratchet. The pushing pawl drives the ratchet down to rotate anticlockwise, and the rotary motion of the ratchet is also transmitted to the main shaft. Figure 4B demonstrates the extension stroke of the EHSA. When the suspension is extended, the slider moves vertically upward and drives the connecting rods and cranks to rotate. At the same time, the pawls move upward. The pushing pawls slide up along the ratchet’s edge without transmitting any motion to the ratchet. The claw-shaped pawls drive the ratchet up to rotate anticlockwise, and the rotary motion of the ratchet is also transmitted to the main shaft.

After such a rectification process, the telescopic motion of the suspension is transformed into the bidirectional vertical motion of the slider and then into the one-way rotation of the ratchet and transmitted to the generator shaft to drive the generator module to output a three-phase current.

#### Generator module

**Figure 5:**
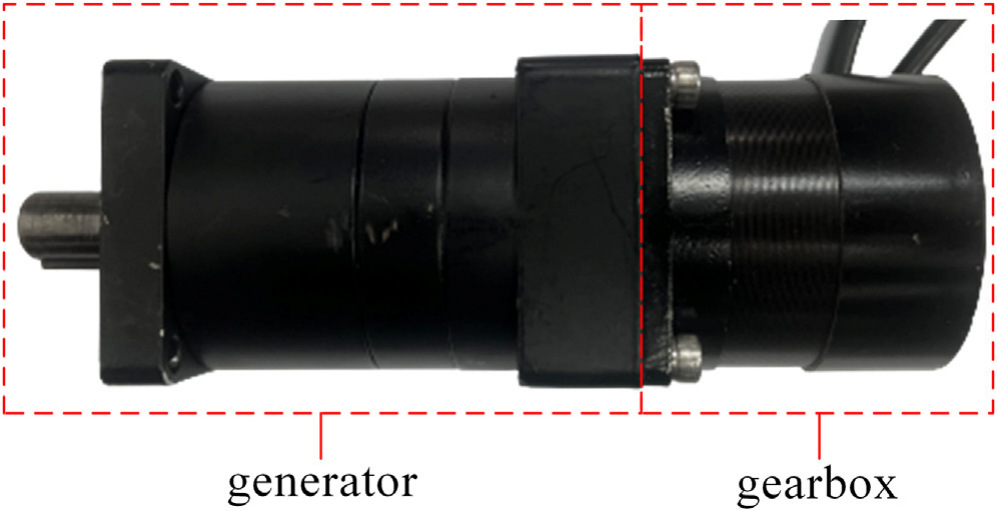
The model and object of the generator module

After the vibration rectification module absorbs and transforms the telescopic motion of freight train suspensions, the one-way rotary motion transferred to the generator module will significantly improve its working conditions, and better power generation performance will be obtained. A matched set of generator and gearbox consists of the generator module, as shown in Figure 5, and is fixedly installed on the base plate by the special bracket. Because of its small rotor inertia, weight, and size, the brushless direct current motor (model 57BL55S06-230TF9) is used as the generator in this study. The gearbox’s transmission ratio is 1:24. Table 1 shows the relevant electrical parameters of this module in detail.

**Table 1:** Relevant electrical parameters of the generator module

Electrical parameters	Value
Rated voltage	24 V
Rated speed	3000 rpm
Rated torque	0.18 N m
Rated power	60 W
Rated current	3.3 A
Back electromotive-force constant	0.04 V s/rad
Torque constant	0.06 N m/A
Internal resistor	1.15 Ω
Number of pole pairs	4
Rotor inertia	7.5 kg mm^2^
Length	166 mm
Width	57 mm
Weight	1.6 kg
Gearing ratio	1:24

#### Energy storage module

**Figure 6:**
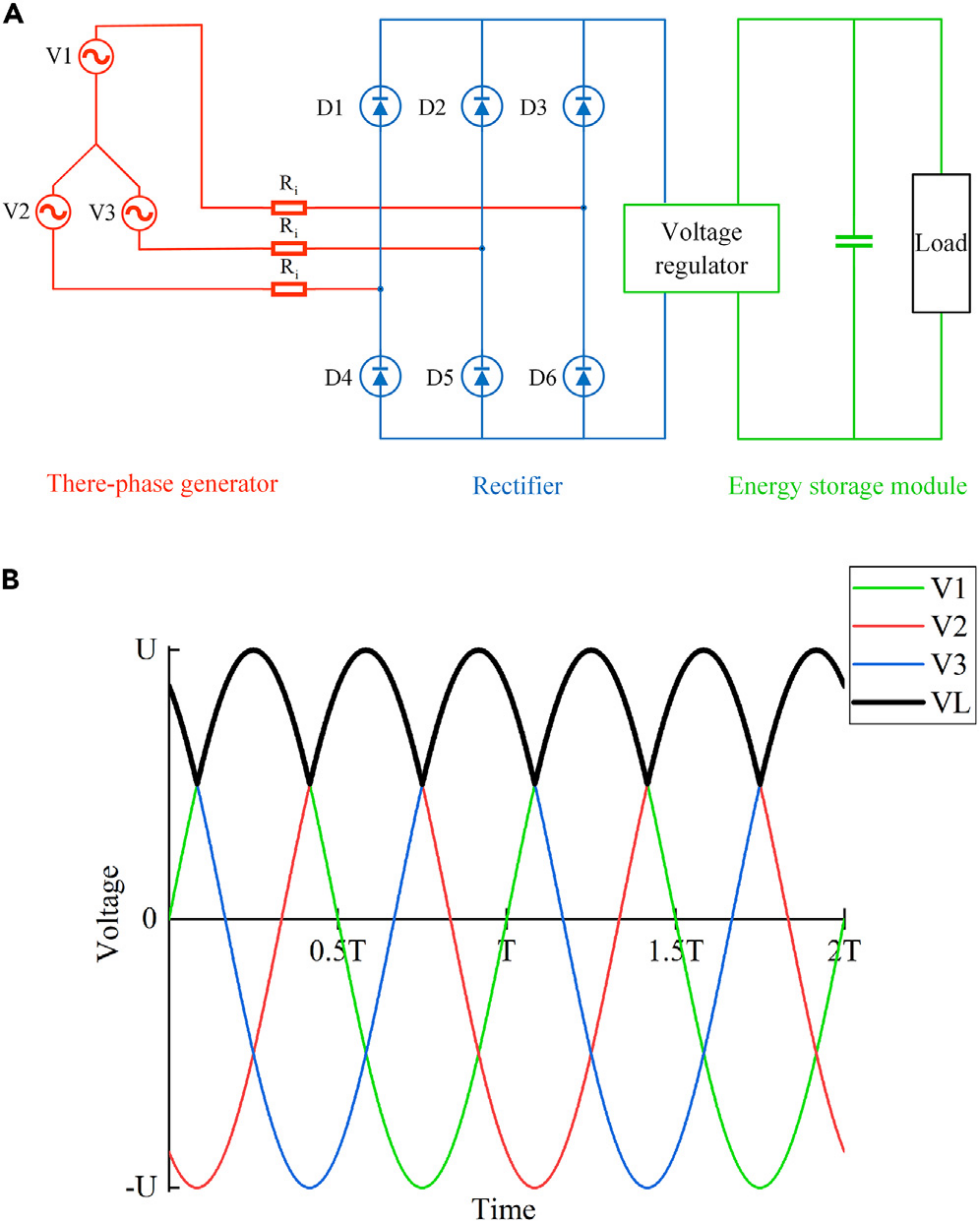
Schematic diagram of the energy storage module (A) Circuit. (B) AC-DC conversion of three-phase rectifier.

The rotational velocity of the main shaft of the generator mainly depends on the vibration frequency of the train suspension. Generally, a freight train’s frequency range of suspension vibration is 1.3Hz–3.5Hz when it travels at a speed of 40–100 km/h.^7^ Since the vibration of freight train suspension is irregular, the voltage and current output from the generator module will fluctuate, making it challenging to maintain a constant value. To solve this problem, supercapacitors are selected to store the energy generated by the EHSA, which have the characteristics of faster charging and discharging higher input and output power and longer working life. Supercapacitors are used in combination with voltage regulators and rectifier circuits to form the complete energy storage module. The circuit schematic diagram is shown in Figure 6A.

The rectifier selected in this paper is a three-phase rectifier, which is a bridge circuit composed of 6 diodes with the unidirectional conduction characteristics. The voltage differences between the phases determine whether each diode is conductive. Figure 6B shows the AC-DC conversion principle of the three-phase rectifier. There is a 120-degree phase difference between the three-phase currents generated by the generator, and the three-phase voltages are represented by V1, V2, and V3 respectively. After rectification, the pulsed direct current will be output, and its voltage is represented by VL.

### Modeling and analysis

In this section, the kinematics of the MMR mechanism in the EHSA is studied first, and then the system’s dynamic model is analyzed. The power generation capacity and damping effect of the EHSA are analyzed based on the system’s dynamic model.

#### Kinematics of the slider-crank mechanism

The vibration rectification module consists of a slider-crank mechanism and a ratchet-pawl mechanism. The slider-crank mechanism converts the reciprocating motion in the vertical direction produced by vibrations in the suspension of freight trains into rotary motion. The ratchet-pawl mechanism restricts the rotary motion to a one-way motion. The suspension is assumed to have sinusoidal harmonic vibration in the vertical direction. Therefore, the motion of the slider fixedly connected to the top of the suspension can be expressed as:

(Equation 1) $$x_{s}=A_{s}\cdot \sin\left(2\pi \cdot f\cdot t\right)$$

where $x_{s}$ is the slider displacement. $A_{s}$, f and t represent the amplitude, the frequency and the time of the sinusoidal harmonic vibration, respectively.

**Figure 7:**
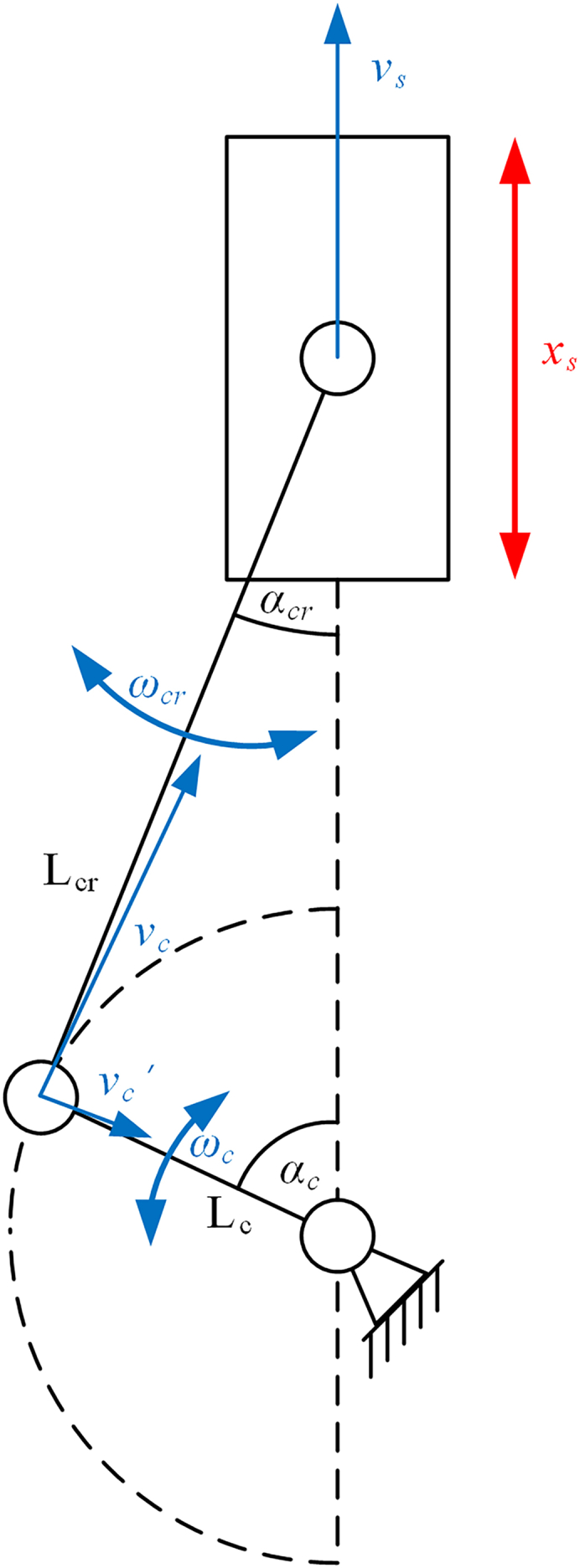
Simplified model of the slider-crank mechanism

As shown in Figure 7, the kinematic relationship is analyzed based on the simplified model of the slider-crank mechanism. Referring to the principle of movement synthesis, the relationship between the slider movement velocity ($v_{s}$) and the movement velocity of the connection point between the connecting rod and the crank ($v_{c}$) can be obtained as follows:

(Equation 2) $$v_{c}=v_{s}+{v_{c}}^{\prime }$$

where ${v_{c}}^{\prime }$ is the velocity at which the connection point rotates around the slider.

According to the geometric relationship between the mechanical parts in Figure 7, the following equation can be obtained:

(Equation 3) $$L_{c}\cdot \sin\;\alpha_{c}=L_{cr}\cdot \sin\;\alpha_{cr}$$

where $L_{c}$ and $L_{cr}$, respectively, represent the lengths of the crank and connecting rod. $\alpha_{c}$ and $\alpha_{cr}$, respectively, represent the rotation angles of the crank and connecting rod.

According to the transformation principle for kinematics, the following formulas can be obtained:

(Equation 4) $$v_{c}=\omega_{c}\cdot L_{c}$$

(Equation 5) $${v_{c}}^{\prime }=\omega_{cr}\cdot L_{cr}$$

(Equation 6) $${v_{c}}^{\prime }\;\cos\;\alpha_{cr}=v_{c}\;\cos\;\alpha_{c}$$

(Equation 7) $$v_{s}=\frac{dx_{s}}{dt}$$

(Equation 8) $$\omega_{c}=\frac{d\alpha_{c}}{dt}$$

where $\omega_{c}$ and $\omega_{cr}$ are the velocities of the crank rotation and connecting rod rotation, respectively.

According to the algorithms of trigonometry, the following formulas can be obtained:

(Equation 9) $$\cos\;\alpha_{cr}=\sqrt{1-\sin^{2}\alpha_{cr}}$$

(Equation 10) $$\sin^{2}\alpha_{c}=\frac{1-\cos\;2\alpha_{c}}{2}$$

By integrating Equations 3, 4, 5, 6, 9, and 10 into Equation 2, after a series of transformations and collation, the detailed expression of the slider movement velocity ($v_{s}$) can be obtained as follows:

(Equation 11) $$v_{s}=\omega_{c}\cdot \left(\frac{{L_{c}}^{2}\cdot \sin\;2\alpha_{c}}{\sqrt{4{L_{cr}}^{2}+2{L_{c}}^{2}\cdot \left(\cos\;2\alpha_{c}-1\right)}}+L_{c}\cdot \sin\;\alpha_{c}\right)$$

By substituting Equations 7 and 8 into Equation 11, the equation with differential factors can be obtained as follows:

(Equation 12) $$A_{s}\cdot \cos\left(2\pi \cdot f\cdot t\right)\cdot 2\pi \cdot f\cdot dt=\left(\frac{{L_{c}}^{2}\cdot \sin\;2\alpha_{c}}{\sqrt{4{L_{cr}}^{2}+2{L_{c}}^{2}\cdot \left(\cos\;2\alpha_{c}-1\right)}}+L_{c}\cdot \sin\;\alpha_{c}\right)\cdot d\alpha_{c}$$

By integrating both sides of Equation 12, the relationship between $\alpha_{c}$ and t can be expressed by the following formula:

(Equation 13) $$A_{s}\cdot \sin\left(2\pi \cdot f\cdot t\right)=-L_{c}\cdot \cos\;\alpha_{c}-\sqrt{{L_{cr}}^{2}-{L_{c}}^{2}\cdot \sin^{2}\alpha_{c}}+C$$

where C is an integral correction constant c.

**Figure 8:**
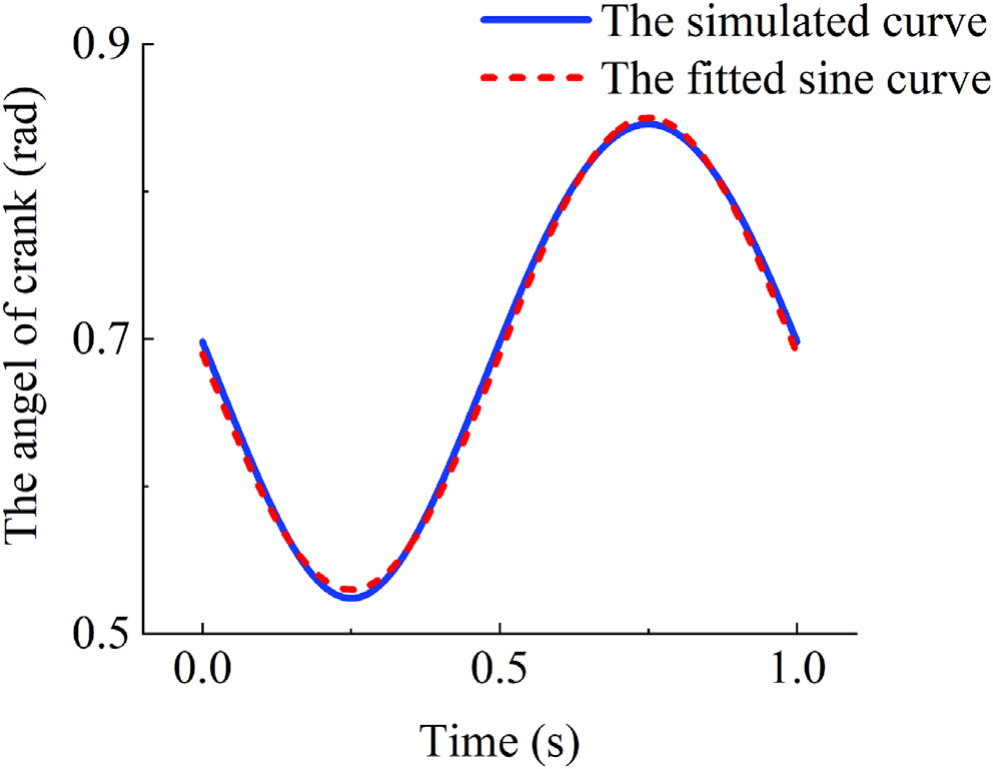
The crank rotation angle curve

By simulating Equation 13 and plotting time-angle curves with MATLAB, it can be seen obviously that the crank can be regarded as a harmonic swing in Figure 8. By fitting the sine curve with MATLAB, the expression of $\alpha_{c}$ can be obtained as follows:

(Equation 14) $$\alpha_{c}=-A_{c}\cdot \sin\left(2\pi \cdot f\cdot t\right)+\alpha_{0}$$

where $A_{c}$ is the amplitude of the harmonic swing, and $\alpha_{0}$ is the initial angle of the crank during the harmonic swing. By simultaneously taking the derivative with respect to time for both sides of Equation 14, the expression of $\omega_{c}$ can be obtained as follows:

(Equation 15) $$\omega_{c}=A_{c}\cdot \cos\left(2\pi \cdot f\cdot t\right)\cdot 2\pi \cdot f$$

Now the proportional relation between the slider movement velocity ($v_{s}$) and the crank rotation velocity ($\omega_{c}$) is defined as follows:

(Equation 16) $$\frac{v_{s}}{\omega_{c}}=k$$

where k is the value of proportion. By integrating Equations 1 and 7 into Equation 16, the expression of k can be obtained as follows:

(Equation 17) $$k=\frac{A_{s}}{A_{c}}$$

After simultaneously taking the derivative with respect to time for both sides of Equation 3, the expression of the velocity of the connecting rod rotation $\omega_{c}$ is as follows:

(Equation 18) $$\omega_{cr}=\frac{L_{c}\cdot \cos\;\alpha_{c}}{L_{cr}\cdot \cos\;\alpha_{cr}}\cdot \omega_{c}$$

Substituting Equations 3 and 9 into Equation 18, it can be seen that the relationship between the crank’s rotational velocity and connecting rod rotation is as follows:

(Equation 19) $$\omega_{cr}=\frac{L_{c}\cdot \cos\;\alpha_{c}}{\sqrt{{L_{cr}}^{2}-{L_{c}}^{2}\cdot \sin^{2}\alpha_{c}}}\cdot \omega_{c}$$

#### Dynamics analysis of the EHSA

**Figure 9:**
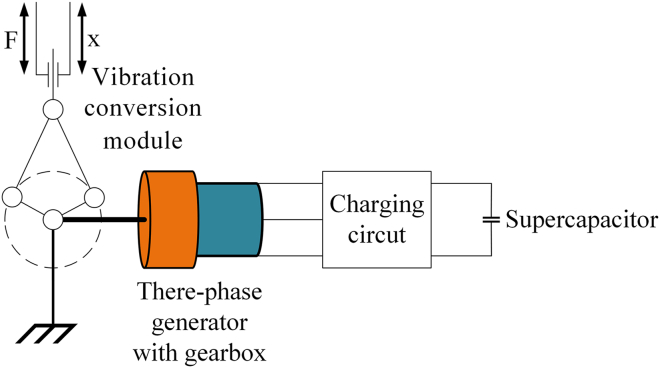
Dynamic model of the EHSA

In order to analyze the dynamic characteristics of EHSA more accurately, the Lagrangian formulation is introduced in this section. The input force F and input displacement x are shown in Figure 9. Easily obtained from the principle of the formulation, in the proposed EHSA, the following dynamic relationship exists:

(Equation 20) $$F=\frac{d}{dt}\left(\frac{\partial T}{\partial \dot{x}}\right)+\frac{\partial E}{\partial x}+\frac{\partial D}{\partial \dot{x}}$$

where F is the excitation force, T is the kinetic energy, $\dot{x}$ is the excitation speed, E is the potential energy, x is the excitation displacement, and D is the damping energy, respectively. For the proposed EHSA, $x=x_{s}$ and $\dot{x}=v_{s}$, so the kinetic energy T is expressed as the following formula:

(Equation 21) $$T=\frac{1}{2}\cdot m_{v}\cdot {v_{s}}^{2}+\frac{1}{2}\cdot \left(4J_{cr}\right)\cdot {\omega_{cr}}^{2}+\frac{1}{2}\cdot \left(4J_{c}\right)\cdot {\omega_{c}}^{2}+\frac{1}{2}\cdot \left(2J_{r}\right)\cdot {\omega_{c}}^{2}+\frac{1}{2}\cdot J_{ms}\cdot {\omega_{c}}^{2}+\frac{1}{2}\cdot J_{gb}\cdot {\omega_{c}}^{2}+\frac{1}{2}\cdot J_{g}\cdot {\omega_{g}}^{2}$$

where $\omega_{g}$ is the generator rotor rotation velocity, $m_{v}$ is the mass of the components moving vertically, $J_{cr}$*,* $J_{c}$*,* $J_{r}$*,* $J_{ms}$*,* $J_{gb}$ and $J_{g}$, respectively, represent the moments of inertia of a single connecting rod, a single crank, a single ratchet, the main shaft, the gearbox and the generator.

For the two other forms of energy E and D, their expressions are given by the following equations:

(Equation 22) $$E=m_{v}\cdot g\cdot x_{s}$$

(Equation 23) $$D=\frac{1}{2}\cdot C_{L}\cdot {v_{s}}^{2}$$

where $C_{L}$ represents the EHSA damping ratio coefficient, which is a linearly varying value.

Figure 9 shows the schematic diagram of the movement of the EHSA, which can be used to quantitatively calculate the value of the damping ratio coefficient of the device. The input power $P_{inp}$ is calculated by the following formula:

(Equation 24) $$P_{inp}=C_{L}\cdot {v_{s}}^{2}$$

From the perspective of energy output, the input power $P_{inp}$ can also be expressed as the following formula:

(Equation 25) $$P_{inp}=P_{g}+P_{f}$$

where $P_{g}$ represents the generator output power, $P_{f}$ is the power consumed due to friction in the slider-crank mechanism, ratchet-pawl mechanism, gearbox, and generator. According to the definition of the generator output power $P_{g}$, it can be expressed as follows:

(Equation 26) $$P_{g}=\eta_{s}\eta_{r}\eta_{gb}\eta_{g}P_{inp}$$

where the efficiency of each component, including the slider-crank mechanism, the ratchet-pawl mechanism, the gearbox, and the generator, is respectively represented by $\eta_{s}$, $\eta_{r}$, $\eta_{gb}$ and $\eta_{g}$.

According to the power balance equation of electrical and mechanical damping, the following relationship can be obtained:

(Equation 27) $$P_{g}=T_{g}\cdot \omega_{g}$$

The following relationship exists between the torque of the generator shaft $T_{g}$ and the generator rotor rotation velocity $\omega_{g}$:

(Equation 28) $$T_{g}=C_{R}\cdot \omega_{g}$$

Equations 27 and 28 are combined to obtain the following relation:

(Equation 29) $$P_{g}=C_{R}\cdot {\omega_{g}}^{2}$$

where $C_{R}$ is the rotation damping ratio coefficient of the EHSA equipped with the circuit, and its expression is as follows:

(Equation 30) $$C_{R}=\frac{1.5\times k_{e}\cdot k_{t}}{R+r}$$

where $k_{e}$ is the back EMF (electromotive force) constant of the generator, $k_{t}$ is the generator torque constant, R is the external load resistance from the circuit, and r is the internal resistance of the generator.

By integrating Equations 24 and 29 into Equation 26, the following formula can be concluded:

(Equation 31) $$C_{R}\cdot {\omega_{g}}^{2}=\eta_{s}\eta_{r}\eta_{gb}\eta_{g}C_{L}{v_{s}}^{2}$$

In the EHSA, the gearbox’s function is to amplify the rotation velocity of the generator rotor. The relationship between the rotational velocity before and after amplification is as follows:

(Equation 32) $$\omega_{g}=i\cdot \omega_{c}$$

where i is the amplification ratio of rotary motion after transmission by the gearbox.

From the reasoning in the previous section, the following equation is known:

(Equation 33) $$v_{s}=k\cdot \omega_{c}$$

By solving Equations 31, 32, and 33 simultaneously, the damping ratio coefficient $C_{L}$ of the EHSA can be expressed by the following formula:

(Equation 34) $$C_{L}=\frac{C_{R}\cdot i^{2}}{\eta_{s}\eta_{r}\eta_{gb}\eta_{g}k^{2}}$$

Then, by replacing some of the factors in Equation 34 with existing relations in Equations 17 and 30, the damping ratio coefficient, $C_{L}$, is written in detail as follows:

(Equation 35) $$C_{L}=\frac{1.5\times k_{e}k_{t}i^{2}}{\eta_{s}\eta_{r}\eta_{gb}\eta_{g}\left(R+r\right){\left(\frac{A_{s}}{A_{c}}\right)}^{2}}$$

By substituting Equations 18, 29, and 31 into Equation 21, The expression of kinetic energy T can be derived in more detail as follows:

(Equation 36) $$T=\frac{1}{2}\cdot \left(m_{v}+\frac{\frac{4J_{cr}{L_{c}}^{2}\;\cos^{2}\alpha_{c}}{{L_{cr}}^{2}\;\cos^{2}\alpha_{cr}}+4J_{c}+2J_{r}+J_{ms}+J_{gb}+i^{2}J_{g}}{{\left(\frac{A_{s}}{A_{c}}\right)}^{2}}\right)\cdot {v_{s}}^{2}$$

Therefore, substituting Equations 22, 23, 35, and 36 into 20 leads to the following:

(Equation 37) $$F=\left(m_{v}+\frac{\frac{4J_{cr}{L_{c}}^{2}\;\cos^{2}\alpha_{c}}{{L_{cr}}^{2}\;\cos^{2}\alpha_{cr}}+4J_{c}+2J_{r}+J_{ms}+J_{gb}+i^{2}J_{g}}{{\left(\frac{A_{s}}{A_{c}}\right)}^{2}}\right)\cdot \frac{dv_{s}}{dt}\pm m_{v}\cdot g+\frac{1.5\times k_{e}k_{t}i^{2}}{\eta_{s}\eta_{r}\eta_{gb}\eta_{g}\left(R+r\right){\left(\frac{A_{s}}{A_{c}}\right)}^{2}}\cdot v_{s}$$

The equivalent inertial mass $m_{ei}$ of the moving parts of the EHSA can be calculated as follows:

(Equation 38) $$m_{ei}=m_{v}+\frac{\frac{4J_{cr}{L_{c}}^{2}\;\cos^{2}\alpha_{c}}{{L_{cr}}^{2}\;\cos^{2}\alpha_{cr}}+4J_{c}+2J_{r}+J_{ms}+J_{gb}+i^{2}J_{g}}{{\left(\frac{A_{s}}{A_{c}}\right)}^{2}}$$

#### Simulation calculation

**Figure 10:**
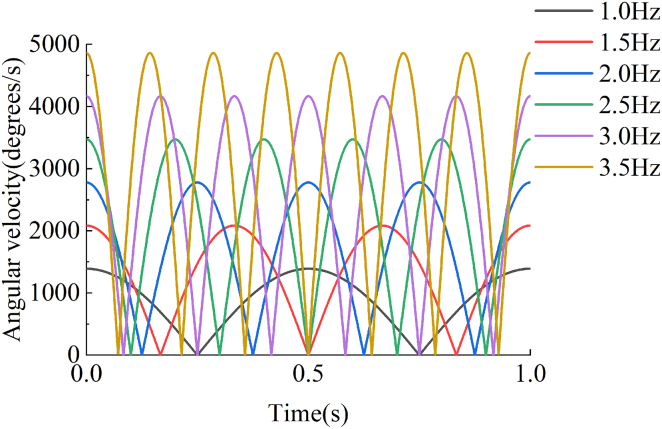
The rotational velocity of the generator rotor at varying frequencies

In order to quantify the specific properties of the EHSA, a corresponding mathematical simulation model is established in MATLAB based on the theoretical kinematics and dynamics of the device, which has been deduced above. Firstly, sinusoidal displacements of different frequencies are input into the mathematical model of the EHSA in simulation. As can be obtained from Equations 15 and 32, the rotational velocity of the generator rotor simulated is shown in Figure 10. Obviously, under the excitation of sinusoidal displacement, the rotational velocity of the generator rotor is proportional to its frequency. The simulated rotational velocities are further verified for the selection of the gearbox and generator, ensuring that the working speed of the generator module is reasonable and efficient.

**Figure 11:**
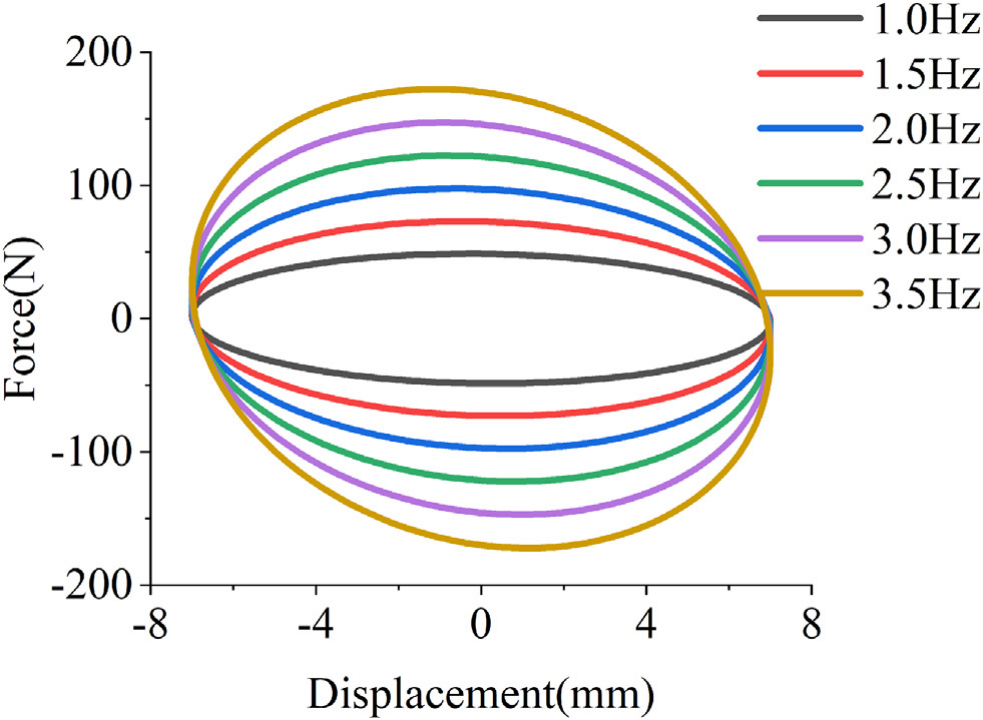
The displacement-force curves in the simulation

Then, before simulating the damping force of the EHSA, it is necessary to estimate the transmission efficiency of each component of the device. In this paper, the transmission efficiency of the slider-crank mechanism, the ratchet-pawl mechanism, the gearbox, and the generator are respectively presented by $\eta_{s}$, $\eta_{r}$, $\eta_{gb}$ and $\eta_{g}$, and their estimated values are respectively 0.93, 0.95, 0.90, and 0.91. Therefore, the overall transmission efficiency of the EHSA is approximately 0.72. Thus, the damping force of the EHSA can be calculated from Equation 37, and the relationship between displacement and force in the simulation is shown in Figure 11. It can be seen that the maximum damping force of EHSA is positively correlated with the excitation frequency when other parameters are unchanged. In addition, due to the mass inertia of ESHA itself, the damping force change trend at any excitation frequency is negative slope.

## Results and discussion

### Bench test details

**Figure 12:**
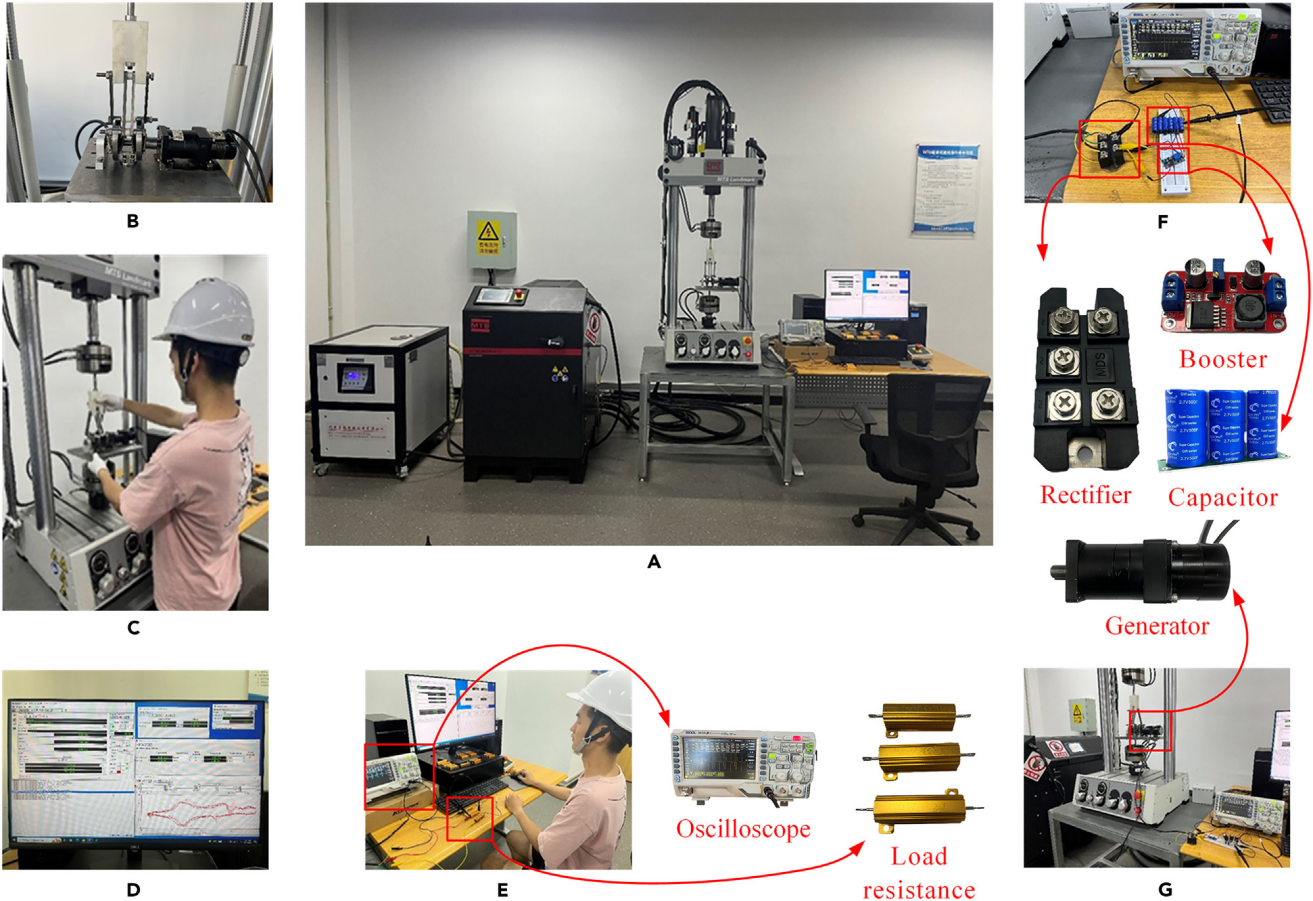
The MTS bench tests of the EHSA (A) Overview. (B) Prototype. (C) Installation. (D) Data recording. (E) Data analysis. (F) Charging circuit. (G) Charging test.

**Figure 13:**
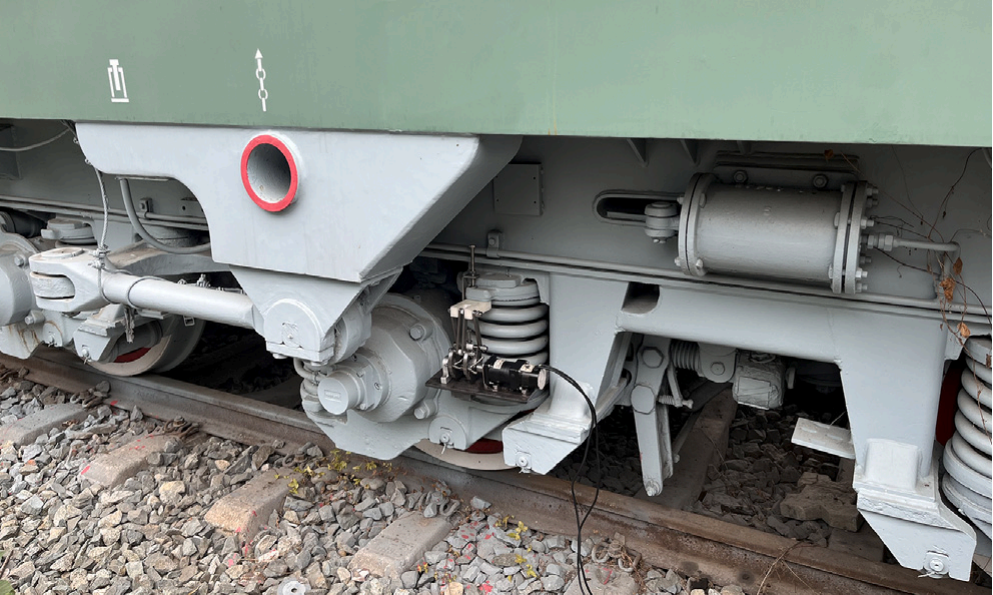
The installation of EHSA on the real freight train

The MTS Landmark Servohydraulic Test System of model 370.02 is selected in this paper, and its relevant technical parameters of the equipment are shown in Table 2. The bench test was carried out using the MTS to measure the performance of the EHSA obtained in simulations, as shown in Figure 12. The overview of setup experiments is shown in Figure 12A, including an MTS system for input sinusoidal displacement and measurement of multiple outputs, an experimental model machine of the EHSA, and a computer. Figure 12B shows the assembled full-scale model machine of the EHSA, and Figure 12C shows its bench installation process. A computer linked to the MTS system can record and save the experimental data of displacement and force received by the sensors in the MTS system, as shown in Figures 12D and 12E shows the preliminary analysis of the experimental data of displacement and force. Figure 12F shows the supercapacitor charging circuits, and the charging test is shown in Figure 12G. In addition to the bench tests, the prototype was installed on a real freight train to verify the universal applicability of the EHSA in freight trains, as shown in Figure 13.

**Table 2:** Relevant technical parameters of the MTS

Technical parameters	Value
Load capacity	±25 kN
Dynamic stroke	100 mm
Measurement frequency	0.1–30 Hz
Measurement accuracy	±0.5%
Maximum vertical measurement space	1335 mm

### Experimental results and analysis

**Figure 14:**
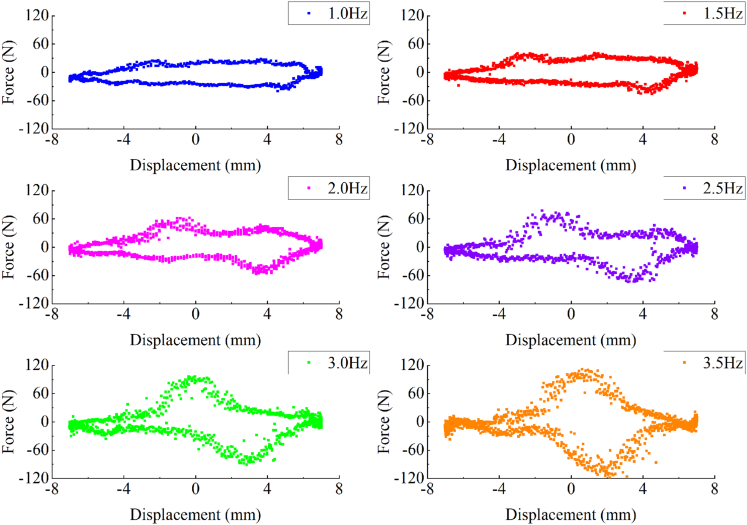
The displacement-force diagram of MTS bench test results for the EHSA

**Figure 15:**
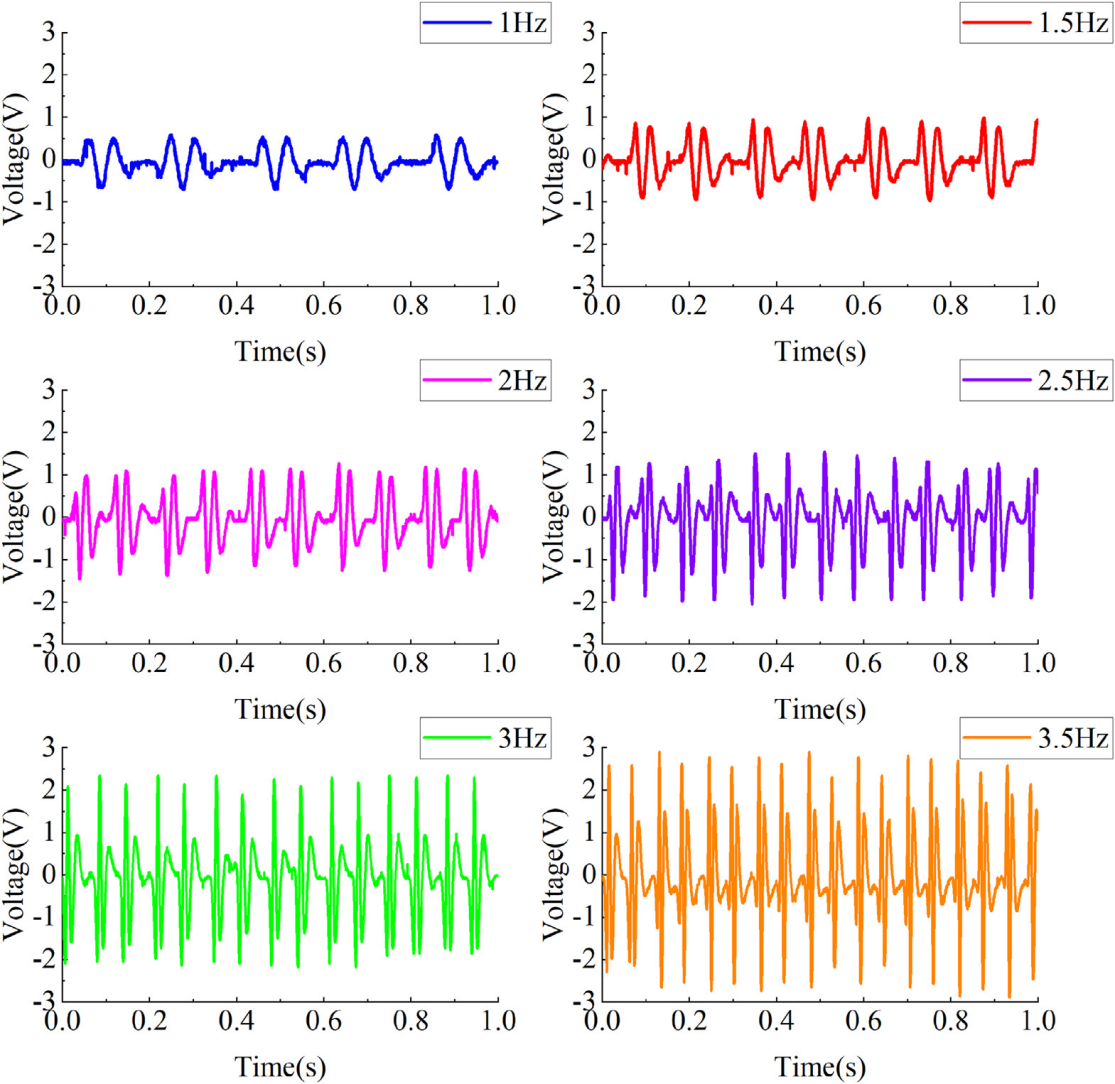
The time-voltage diagram of MTS bench test results for the EHSA

The main parameters that need to be set in the experiments are the mechanical parameters, which include: the amplitude of excitation displacement is 7mm; the excitation frequencies are 1Hz, 1.5Hz, 2Hz, 2.5Hz, 3Hz, and 3.5Hz, respectively. In addition, supercapacitors with capacities of 1mF, 2mF, and 3mF are selected in the charging tests of this experiment. Figures 14 and 15 show the experimental results of the EHSA with the rated load on the MTS bench.

In detail, Figure 14 is the force and displacement data collected by the MTS system. It is known that the displacement-force annular area represents the vibration energy absorbed by EHSA in a period. The results of bench tests show that the maximum damping force of EHSA is positively correlated with the excitation frequency, and the energy storage effect of EHSA is improved with the increase of excitation frequency. It is worth pointing out that, with the increased frequency, the moving parts in the EHSA will be affected by the more obvious flywheel effect.^27^ Moreover, it is obvious that when the moving parts of the EHSA move near the extreme position, the magnitude of the force is close to none. Based on the mentioned conditions, the displacement-force data recorded in the experiments are shown in Figure 14.

Figure 15 is the voltage data collected by the oscilloscope in this experiment. According to the analysis of Figure 15, when the amplitude of the input sinusoidal displacement is 7mm, and the rated value of the selected load resistance is 4Ω, with the increase of the frequency of the input sinusoidal displacement, the resulting voltage waveforms are similar, but the maximum voltage and the frequency of the voltage waveform output from the EHSA increase.

These data obtained from the MTS system were mutually verified with the results of the simulation experiments in the section modeling and analysis, which also confirmed the generating capacity of the EHSA.

The power input $P_{inp}$ by the MTS system to the EHSA can be calculated by the following formula:

(Equation 39) $$P_{inp}=\frac{\Delta W}{T}$$

where ΔW is the total amount of mechanical energy input by the MTS system, whose value is obtained by integrating displacement and force data in Figure 14, and T is the periodic value of sinusoidal displacement input by the MTS system.

The power output $P_{o}$ from the EHSA can be calculated by the following formula:

(Equation 40) $$P_{o}=\frac{3\times \int_{0}^{T}\left(V^{2}\left(t\right)/R\right)dt}{T}$$

where V(t) represents the value of the voltage across the load resistor.

In the bench test, the overall efficiency η of the EHSA can be calculated by the following formula:

(Equation 41) $$\eta =\frac{P_{o}}{P_{inp}}$$

For the mechanical transmission efficiency $\eta_{m}$ of EHSA, its value can be obtained by the following formula:

(Equation 42) $$\eta_{m}=\frac{\eta }{\eta_{c}}$$

where $\eta_{c}$ is the efficiency of the circuit, which can be calculated by substituting the initial conditions of the experiments into the following formula:

(Equation 43) $$\eta_{c}=\frac{R}{R+r}$$

Table 3 shows the average power input from the MTS, the average power output by the EHSA, the overall generation efficiency, and the mechanical transmission efficiency of the EHSA under different excitation frequencies. It can be revealed from Table 3 that the mechanical transmission efficiency of the EHSA presents a positive correlation with the excitation frequency.

**Table 3:** Experimental result values at different frequencies

Input frequency	P_inp_	P_o_	η	η_m_
1 Hz	0.50W	0.13W	26.35%	34.58%
1.5 Hz	0.88W	0.28W	32.30%	42.39%
2 Hz	1.32W	0.48W	36.58%	48.01%
2.5 Hz	1.83W	0.77W	42.03%	55.17%
3 Hz	2.44W	1.18W	48.33%	63.43%
3.5 Hz	3.19W	1.65W	51.62%	67.75%

Luigi Costanzo et al. proposed an electronic interface for the maximization of the power extraction from train suspension energy harvesters. The proposed novel maximum power point tracking (MPPT) technique is called speed drive adaptive (SDA), which makes the output voltage stable at the optimal value quickly by using the measurement of the generator speed, and is not affected by the time-varying of the train suspension vibrations. SDA technique also enables dynamic adaptive control to ensure maximum power output under system tolerances and time-varying conditions. Compared with the current widely used Perturb and Observe (P&O) technique, SDA technique has better performance.^28^ In subsequent articles, Luigi Costanzo et al. focused on AC-DC converters for train suspension energy harvesters. For the diode bridge rectifier, the proposed speed-voltage model is very accurate in predicting DC voltage output by generators with constant speed or time-varying speed, which can provide effective assistance in the design of high-performance train suspension energy harvesters equipped with MPPT technique.^29^ The EHSA can obtain better power generation effect and efficiency both theoretically and experimentally if the optimization of the above techniques is adopted in the energy storage module.

**Figure 16:**
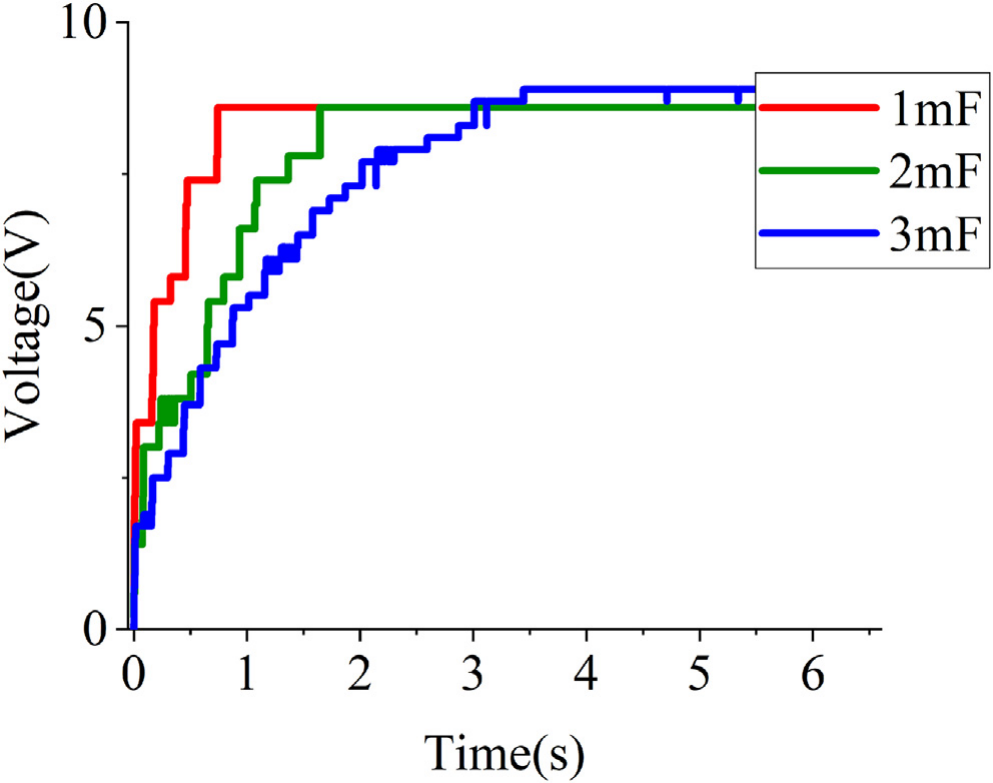
Charging test of the supercapacitors with different capacity

Figure 16 shows the charging test results of the EHSA on the MTS bench. These results include the charging voltages of supercapacitors with different capacities when the frequency of the sinusoidal excitation is 2Hz, and the amplitude is 7mm. It can be seen that under this excitation, the output voltage of the EHSA is around 8.7V.

In the charging test, because only one rated sinusoidal excitation is set, the voltage output by the EHSA is the same, and theoretically the supercapacitor should also be charged to the same voltage. According to Figure 16, it can be seen that the voltages of the supercapacitors during the charging process rise rapidly and then gradually become constant but not exactly the same, this is because the selected supercapacitors have small capacities and are more sensitive to the impedance existing in each component in the circuit, resulting in some experimental errors, but this does not affect the conclusions obtained. In addition, as the capacity of the supercapacitor increases, the time it takes to be charged to the ultimate voltage also increases. This is because the two poles of the supercapacitor with a larger capacity can hold more positive and negative charges respectively, so it takes more time to reach the constant voltage. It should be noted that supercapacitors are particularly suitable for use with on-board self-powered equipment due to their short charging time and wide range of optional capacities.

In order to reflect the improvement in power generation performance of the proposed EHSA, the existing on-board train electromagnetic energy harvesters are summarized in Table 4, in which all the data are from the corresponding literature, and in which their characteristics, installation positions and power generation performance are introduced and compared. It can be seen from the comparison that the proposed EHSA has higher mechanical efficiency.

**Table 4:** Comparison of on-board train electromagnetic energy harvesters

No	Reference	Main characteristics	Installation position	Input	Performance		
					Voltage	Power	Efficiency
1	De Pasquale et al.^30^	Magnetic suspended proof mass (simple structure and small package)	Wheelset axle box	80 km/h	2.5 V (Vmax)	100 mW (Pmax)	/
2	Ung et al.^31^	2-DoF vibration-energy-harvesting device (generating energy for the unloaded and loaded travel condition)	Suspension	3.92 m/s^−2^	/	200 mW (Pmax)	/
3	Manoach et al.^32^	Non-contact and non-magnetic mechanical spring (suitable for magnetic environment and better robustness)	Wagon	27 Hz	1.7 V (Vp-p)	10 mW (Pmax)	/
4	Pan et al.^33^	Specifically designed enclosed gearbox and two-guide rail structure (greater efficiency and reliability)	Suspension	90 km/h (bench); 30 km/h (on-board)	/	14.5W (Pavg); 73.2W (Pmax)	/
5	Gao et al.^7^	The gear ring mechanism (the compact size and the collection of the vibration energy in multiple directions)	Bogie	80 km/h	1.45 V (Vrms)	263 mW (Prms)	65%
6	Perez et al.^34^	2-DOF energy harvester (significantly increased harvested electrical power)	Bogie	40 Hz	/	6.5 mW (Pavg)	/
7	Li et al.^23^	Energy regeneration shock absorber (generating power while providing suitable damping for train suspensions)	Suspension	3Hz, 12.5mm		24.6W (Pmax)	56.4%
8	Costanzo et al.^28^	power electronic interface equipped with the SDA MPPT technique (adaptive control and better performance)	Suspension	2Hz, 3mm	/	7.3W (Pavg)	/
9	Dong et al.^35^	Energy-regenerative shock absorber (adjustable system damping and power)	Bogie	1.8Hz, 1.35mm	/	9W (Pmax)	/
10	This work	Energy harvesting shock absorber (Easy installation, reliable transmission, high power generation and mechanical efficiency)	Suspension	3.5Hz, 7mm	8.9V (Vmax)	1.65W (Pmax)	67.75%

### Conclusions

In this paper, an energy-harvesting shock absorber for freight trains is proposed to convert the vibration energy of freight trains into electrical energy. The device can be used to provide a continuous and stable power supply for on-board sensors, monitors, and controllers, which can timely detect the running status of the freight trains and ensure the safety of the freight trains. The design of the device is based on the slider-crank mechanism and ratchet-pawl mechanism, and the theoretical analysis, simulation testing, modeling, manufacturing, installation, and experimental testing are also carried out in this paper. Among them, the theoretical analysis includes the kinematics of the MMR mechanism and the dynamics of the whole system, and the simulation test mainly includes the mechanical properties of the device. Then, in the MTS bench test, when the excitation amplitude was set at 7mm, the frequency at 3.5Hz, and load resistance at 4Ω, the measured power generation performance of the device reached the highest, that is, the mechanical efficiency was 67.75%, and the output power was 1.65W. Finally, the charging test proves that the device is suitable for use with on-board self-powered equipment.

### Limitations of the study

Further optimization and field testing are still required for the proposed vibration energy harvesting shock absorber. The limitations of this study can be summarized as follows.

(1) Due to the limitations of experimental conditions and equipment, both simulation and experimentation utilized regular sine wave excitation. To accurately assess the power generation performance of the proposed device on a real train, future studies will involve tests conducted on operational freight trains.
(2) By optimizing material selection and enhancing the accuracy of processing and assembly, the mechanical efficiency and durability of the proposed device will be further improved in the future.
(3) The current study concentrates on the mechanical structure and efficiency of the power generation process. Future enhancements include the incorporation of a power optimization circuit to enhance overall power generation efficiency.

## STAR★Methods

### Key resources table

**Table:** 

REAGENT or RESOURCE	SOURCE	IDENTIFIER
**Software and algorithms**		

Microsoft Visio 2019	Microsoft	https://www.microsoft.com/zh-cn/microsoft-365/visio/flowchart-software
MATLAB 2022a	Mathworks	https://www.mathworks.com/products/matlab.html

**Other**		

Landmark 370 servo-hydraulic test system	Mechanical Testing and Sensing (MTS) Systems Corporation	https://www.mts.com/en/products/materials/dynamic-materials-test-systems/landmark-servohydraulic
DS1102Z-E digital oscilloscope	RIGOL	https://rigol.com

### Resource availability

#### Lead contact

Further information and requests for resources and reagents should be directed to and will be fulfilled by the lead contact Dabing Luo (dbluo@swjtu.edu.cn).

#### Materials availability

This study did not generate new unique reagents.

### Method details

All methods can be found in the main text. Please check the System design section for the design details of the system. Please check the Dynamic analysis and simulation section for the simulation and analysis details of the system. Please refer to the Experimental details section for details of the experimental design and equipment performance parameters.

Microsoft Visio 2019 is used to generate the visual images in the manuscript. MATLAB 2022a is used to process experimental data and generate visual images in the manuscript. A demo video is provided to introduce the whole article more vividly.

### Quantification and statistical analysis

Microsoft Visio 2019 is used to generate the visual images in the manuscript. The voltage signals are captured by the DS1102Z-E digital oscilloscope. The force-displacement signals are captured by the force sensor and displacement sensors integrated into the Landmark 370 servo-hydraulic test system at a sampling frequency of 300 Hz. MATLAB 2022a is used to process experimental data and generate visual images in the manuscript. Through MATLAB 2022a, the voltage signal and force-displacement data are processed to analyze the input and output characteristics of the system.

## Data Availability

• All data reported in this paper will be shared by the lead contact upon reasonable request. • This paper does not report the original code. • Any additional information required to reanalyze the data reported in this paper is available from the lead contact upon request.
